# Spatiotemporal changes in regularity of gamma oscillations contribute to focal ictogenesis

**DOI:** 10.1038/s41598-017-09931-6

**Published:** 2017-08-24

**Authors:** Yosuke Sato, Simeon M. Wong, Yasushi Iimura, Ayako Ochi, Sam M. Doesburg, Hiroshi Otsubo

**Affiliations:** 1 Division of Neurology, Hospital for Sick Children, Toronto, Ontario Canada; 2 Department of Neurosurgery, Showa University School of Medicine, Tokyo, Japan; 3 Department of Diagnostic Imaging, Hospital for Sick Children, Toronto, Ontario Canada; 4 Department of Biomedical Physiology and Kinesiology, Simon Fraser University, Burnaby, British Columbia Canada

## Abstract

In focal ictogenesis, gamma oscillations (30–70 Hz) recorded by electroencephalography (EEG) are related to the epileptiform synchronization of interneurons that links the seizure onset zone (SOZ) to the surrounding epileptogenic zone. We hypothesized that the synchronization of interneurons could be detected as changes in the regularity of gamma oscillation rhythmicity. We used multiscale entropy (MSE) analysis, which can quantify the regularity of EEG rhythmicity, to investigate how the regularity of gamma oscillations changes over the course of a seizure event. We analyzed intracranial EEG data from 13 pediatric patients with focal cortical dysplasia. The MSE analysis revealed the following characteristic changes of MSE score (gamma oscillations): (1) during the interictal periods, the lowest MSE score (the most regular gamma oscillations) was always found in the SOZ; (2) during the preictal periods, the SOZ became more similar to the epileptogenic zone as the MSE score increased in the SOZ (gamma oscillations became less regular in the SOZ); and (3) during the ictal periods, a decreasing MSE score (highly regular gamma oscillations) propagated over the epileptogenic zone. These spatiotemporal changes in regularity of gamma oscillations constitute an important demonstration that focal ictogenesis is caused by dynamic changes in interneuron synchronization.

## Introduction

Focal seizures are generated when the synchronization of interneuronal activity, which is enhanced during interictal periods^1–3^, declines and causes the collapse of the excitation/inhibition balance in preictal periods^4–6^, and is once more intensified in ictal periods^7–10^. In epilepsy caused by focal cortical dysplasia (FCD), GABAergic activity that is intrinsically generated by abnormal interneurons in FCD tissue^11, 12^ contributes to the synchronization of the epileptogenic network, leading to focal seizure generation (ictogenesis)^13, 14^. A recent study in human neocortical epilepsy demonstrated that ictogenesis is related to a dynamic imbalance in synchronization between the seizure onset zone (SOZ) and the surrounding epileptogenic zone^15^. Dehghani *et al*.^16^ showed that the excitatory and inhibitory neurons are tightly balanced across all states of the wake-sleep cycle in both human and monkey, but this balance breaks down during siezures^16^.Our group also reported that the epileptogenic zone in patients with FCD type II exhibits a logarithmic decrease in high frequency (>30 Hz) interregional synchronous connectivity during seizure initiation and propagation^17^.

The topography of high-frequency oscillations (HFOs; >80 Hz) is strongly related to the epileptogenic zone in the human neocortex^18^. Although the removal of cortical regions expressing HFOs is associated with a good postsurgical outcome^19–21^, HFOs identified during presurgical mapping may also include naturally occurring HFOs, and thus not represent true epileptogenic tissue^22, 23^. Alvarado-Rojas *et al*.^24^ recently reported that interictal HFOs (150–200 Hz) were associated with rhythmic inhibitory GABAergic signaling, while preictal HFOs were linked to a failure of GABAergic signaling^24^.

Computer^25–28^ and experimental^29–31^ models of focal seizures have established that GABAergic activity also contributes to the generation of gamma oscillations (30–80 Hz). Grasse *et al*.^32^ reported that synchronous interneuron activity, which become coherent with local field oscillations at gamma band, is a hall mark during the transition from interictal to ictal states in the pirocarpine rat model of epilepsy^32^. Other studies have demonstrated that regular and synchronous gamma oscillations are crucial for synchronizing networks of interneurons in the human cortex^33–35^, and that they are present in the human SOZ^36, 37^. Another study has suggested that rhythmicity of electroencephalographic (EEG) oscillations correlates with focal neuronal synchronization during seizure^38^. Accordingly, interneuron synchronization (primarily GABAergic) can be measured by quantifying gamma oscillation rhythmicity.

Whereas gamma oscillations and HFOs are both related to the generation of epileptic seizures, HFOs are inherently transitory, and thus less likely to embody metastable network states that govern the transitions among interictal, preictal, and ictal states. By contrast, gamma oscillations tend to be ongoing, and are thus in a position to modulate the background cortical rhythmicity that contributes to the cortical state. Therefore, measuring gamma oscillations would enable the better definition of the boundaries of epileptogenic tissue, potentially to improve surgical outcomes. The multiscale entropy (MSE) is a powerful tool that can quantify the dynamic complexity of physiological signals on different time scales^39^ and provide useful insights into the network-controlling mechanisms that underlie physiological dynamics^40^. In neuroscience, MSE analysis has been recently used to analyze background scalp EEG activity in schizophrenia^41^, Alzheimer’s disease^42^, and absence seizures^43^. Because accumulating evidence suggests that gamma oscillations are vital for the dynamics and metastability of both physiological and pathological neocortical networks, we used MSE analysis to evaluate the regularity of gamma oscillations obtained from intracranial EEG.

Here we hypothesized that the synchronization of interneurons could be detected as changes in the regularity of gamma oscillation rhythmicity by using MSE analysis. This study was also driven by a desire to find accurate markers for the SOZ, the epileptogenic zone, and ictal onset. To determine the spatiotemporal dynamics of gamma oscillation rhythmicity across the interictal, preictal, and ictal periods, we calculated the MSE scores in gamma oscillations (28.6–60.7 Hz) from individual intracranial electrodes during each time period in the SOZ, the resection area (RA) outside the SOZ (RA-SOZ), and outside the RA. This is the first report to characterize spatiotemporal changes in the MSE score of gamma oscillations that are associated with focal ictogenesis in patients with FCD type II.

## Results

### Changes in MSE score among three interictal epochs

To assess the stability of interictal EEG data, we compared three 20-s interictal epochs (during non-REM sleep and separated from seizure activity by at least 1 h) using Bartlett’s test and one-way analysis of variance, and confirmed that there were no significant differences between the MSE score variances (p > 0.05) and the MSE scores (p > 0.05) among three interictal epochs per patient.

### Changes in MSE score from interictal to ictal periods for each patient

Overall, we analyzed EEG data (thirty-nine 20-s epochs) recorded from 1164 electrodes (mean ± SD; 90 ± 15 per patient). The number of electrodes whose data were analyzed is summarized for each patient by region in Table 1.

**Table 1:** Clinical characteristics of participating patients with FCD type II.

Patient	Gender	Age (years)		FCD location	Number of electrodes			Ratio of RA to total analyzed area RA/(RA + Outside RA)
		At onset	At surgery		SOZ	RA-SOZ	Outside RA	
1	F	2	13	Rt. frontal	2	34	68	0.35
2	M	3	15	Rt. frontal	1	17	50	0.26
3	M	1	3	Rt. perirolandic	2	33	47	0.43
4	M	3	4	Rt. occipital	1	37	62	0.38
5	M	1	12	Lt. temporal	1	44	56	0.45
6	M	13	15	Lt. perirolandic	1	46	54	0.47
7	F	3	12	Lt. perirolandic	2	26	77	0.27
8	M	5	7	Lt. frontal	2	5	102	0.06
9	F	10	14	Rt. parieto-occipital	1	14	62	0.19
10	M	2	3	Lt. perirolandic	1	19	44	0.31
11	F	2	3	Rt. frontal	1	19	70	0.22
12	F	2	4	Lt. frontal	1	11	87	0.12
13	M	8	9	Lt. frontal	1	3	60	0.06

*F*, female; *FCD*, focal cortical dysplasia; *Lt*., left; *M*, male; *RA*, resection area; *Rt*., right; *SOZ*, seizure onset zone.

**Figure 1:**
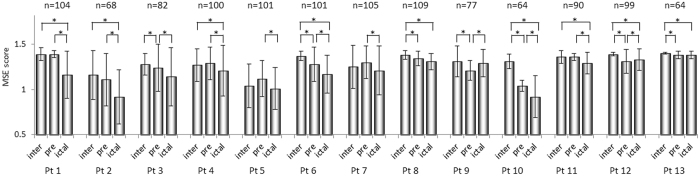
Comparison of multiscale entropy (MSE) score in the gamma band among interictal (inter), preictal (pre), and ictal periods for each patient (Pt). The results are plotted as the mean ± standard deviation (error bar). n represents the number of electrodes analyzed for each patient. Significant differences were evaluated with a Steel-Dwass test. P-values < 0.05 were considered significant and are denoted by an asterisk (*).

**Figure 2:**
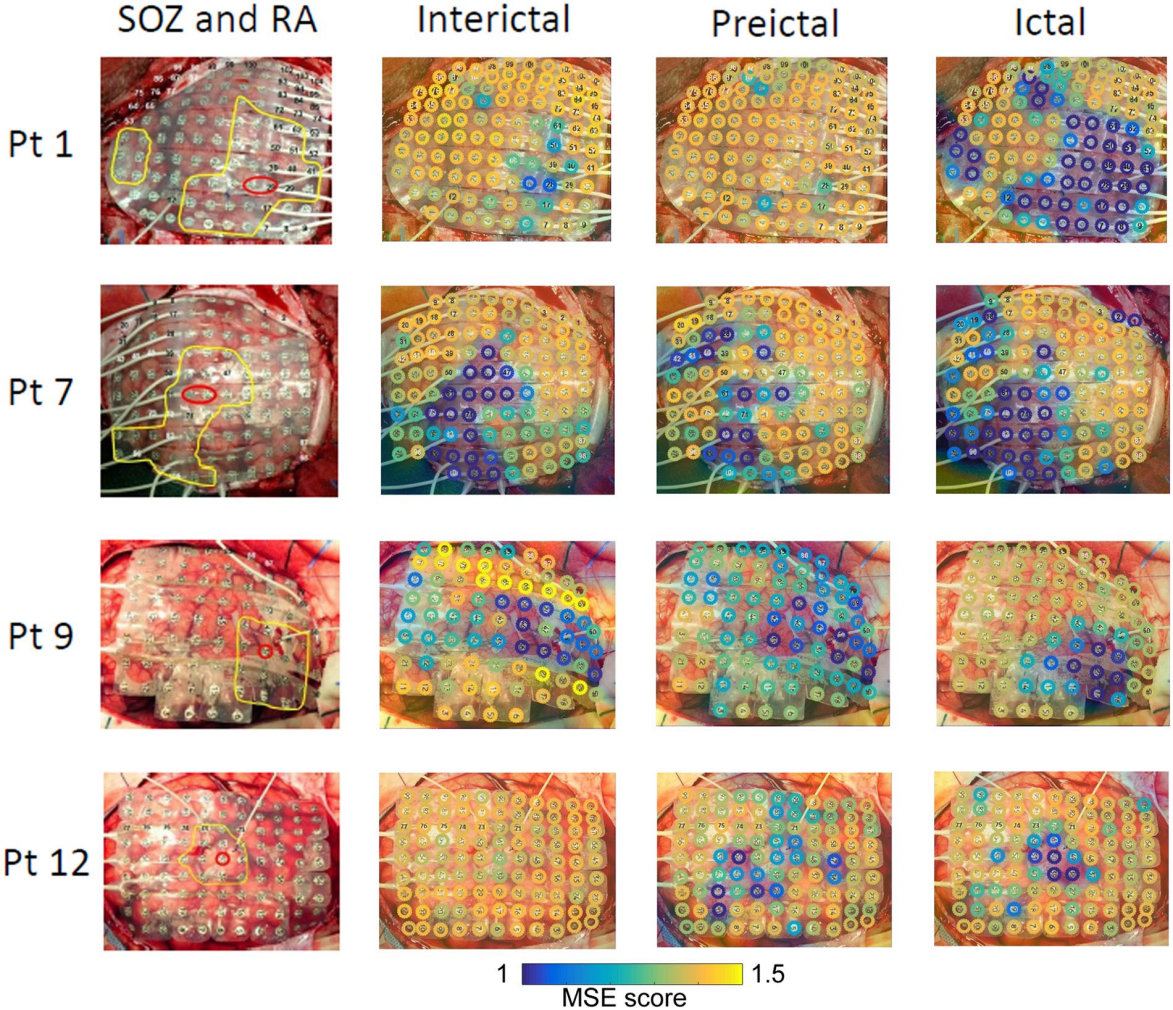
Topographic maps of multiscale entropy (MSE) scores in four representative patients (Pt 1, 7, 9, and 12). The leftmost column shows the location of the seizure onset zone (SOZ; red circles) and the resection area (RA; outlined in yellow). The other 3 columns (from left to right) represent interictal, preictal, and ictal MSE-score maps. Color-coded MSE scores are topographically superimposed onto an intraoperative photograph of each individual patient’s brain surface and grid.

As shown in Fig. 1, the overall MSE score decreased significantly from the interictal to preictal periods in 7 patients, from the preictal to ictal periods in 8 patients, and from the interictal to ictal periods in 9 patients. Among the four patients with the lowest ratios of RA to total analyzed area (meaning those for whom the MSE scores changed mainly outside the RA; Table 1), two showed a significant increase in MSE scores from the preictal to ictal periods (Pt 9 and 12 in Fig. 2) and two showed no change (Pt 8 and 13). These results indicate that MSE scores might be especially low inside the RA during ictal periods.

### Spatiotemporal changes in MSE score

**Figure 3:**
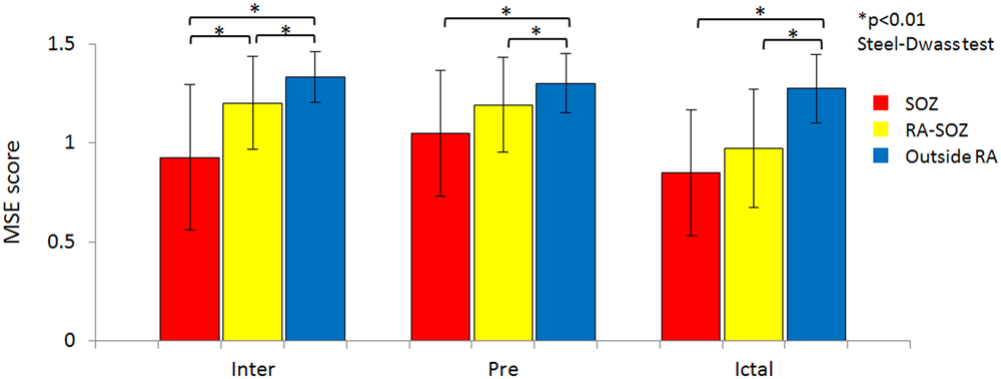
Differences in multiscale entropy (MSE) scores among the three areas for each period. The error bars indicate mean ± standard deviation. MSE scores were compared with Steel-Dwass tests. P-values < 0.01 were considered significant and are denoted by an asterisk (*). Inter, interictal; Pre, preictal; SOZ, seizure onset zone; RA-SOZ, resection area outside the SOZ; Outside RA, outside resection area.

Topographic maps of MSE scores (Fig. 2) indicated that, for each patient, the SOZ was always the site of the lowest MSE scores during the interinctal period. The topographies of lower MSE scores during preictal periods tended to be scattered, but were confined to the RA during ictal periods. Interictal MSE scores were significantly lower in the SOZ (mean ± SD: 0.93 ± 0.37) than in the RA-SOZ (1.20 ± 0.23) or outside the RA (1.33 ± 0.13) (Fig. 3, both p < 0.01), and scores in the RA-SOZ were significantly lower than those outside the RA (p < 0.01). Preictal scores showed a similar pattern in that scores in the SOZ (1.05 ± 0.32) were significantly lower than those outside the RA (1.30 ± 0.15) (p < 0.01), however they did not differ significantly from those in the RA-SOZ (1.19 ± 0.24). Ictal scores were much lower both in the SOZ (0.85 ± 0.32) and RA-SOZ (0.97 ± 0.30) than outside the RA (1.27 ± 0.17) (both p < 0.01).

Taken together, we found that (1) during the interictal periods, the lowest MSE score (the most regular gamma oscillations) was always found in the SOZ; (2) during the preictal periods, the SOZ became more similar to the epileptogenic zone as the MSE score increased in the SOZ (gamma oscillations became less regular in the SOZ); and (3) during the ictal periods, a decreasing MSE score (highly regular gamma oscillations) propagated over the epileptogenic zone. Accordingly, we expect that the interictal low MSE score can act as a useful clinical marker for the SOZ, that the preictal increasing MSE score in the SOZ can for the timing of ictal onset, and that the ictal low MSE score can for the epileptogenic zone.

## Discussion

**Figure 4:**
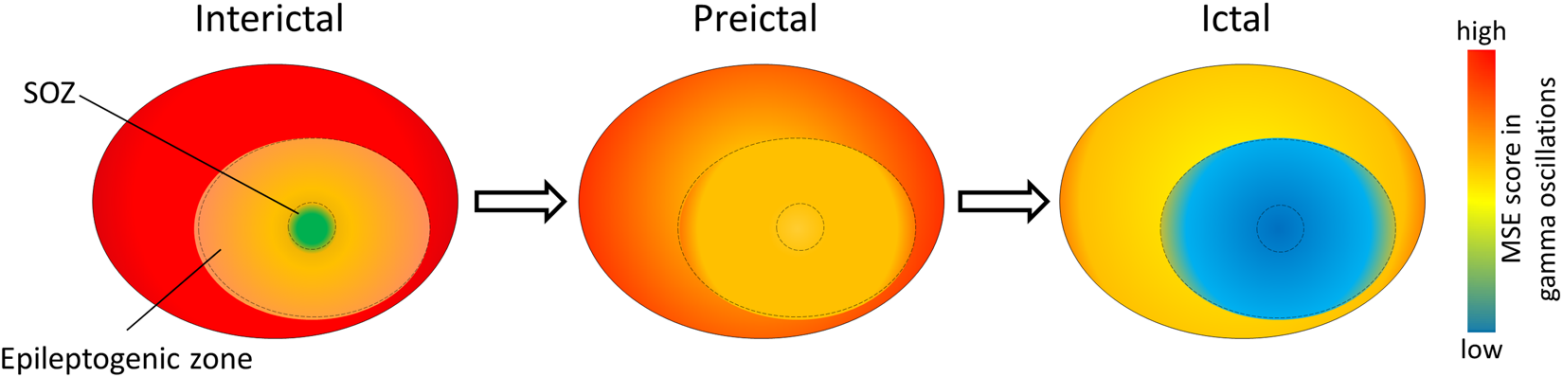
Schematic representation of the spatiotemporal dynamics of the multiscale entropy (MSE) score in gamma oscillations during the transitions leading to an ictal period. Our results suggest that (1) during interictal periods, inhibitory interneuron synchronization (low MSE score) is maintained at high levels in the seizure onset zone (SOZ), while the desynchronizing (high MSE score) effect that acts to prevent seizures is maintained in the surrounding zones; (2) the preictal periods begin with the collapse of the balance between interneuron inhibition and excitation (increasing MSE score in the SOZ), which leads epileptic activity to assimilate the SOZ into the epileptogenic zone; and (3) In ictal periods, hypersynchronization (decreasing MSE score) via excitatory interneuron networks propagates over the epileptogenic zone to precipitate the seizure.

As gamma oscillation rhythmicity strongly reflects synchronization of interneurons^36–38^, we interpreted the observed MSE dynamics as follows: (1) during interictal periods, inhibitory synchronization was excessive in the SOZ, while a desynchronizing effect, which acts to prevent seizures, was preserved in the surrounding zones; (2) the collapse of the excitation/inhibition balance (increasing MSE score in the SOZ) led epileptic activity to assimilate the SOZ into the epileptogenic zone during preictal periods; and (3) the hypersynchronization (decreasing MSE score) via excitatory interneuron networks propagated over the epileptogenic zone to precipitate the seizure during the ictal periods (Fig. 4).

A large-scale network analysis revealed that coherent gamma oscillations were generated through increases in spiking synchrony within local groups of cortical neurons^27^. Additionally, local interneuron connectivity can explain the long-range synchrony of gamma oscillations^28^. Synchronous GABA_A_ receptor-dependent activity is known to play a pivotal role in the focal ictogenesis observed in neuronal networks of FCD^11–14^. Here we show that inhibitory synchronization in the SOZ that was maintained throughout interictal periods was expressed as very low MSE scores. Khambhati *et al*.^15^ reported that seizures are generated by the break down in the synchronous relationships that isolate the SOZ from the surrounding epileptogenic zone^15^. Ibrahim *et al*.^17^ also demonstrated that altered network synchronization, including altered gamma oscillations, plays a role in the initiation and propagation of seizures^17^. Given these evidences, the efficacy of local inhibition that is mediated by hypersynchronization of interneuron networks may play a role in isolating the SOZ during interictal periods.

A study performed on postsurgical tissue from human temporal lobe epilepsy revealed that interictal discharges (IIDs) were preceded by interneuronal firing that depended on glutamatergic and GABAergic activity, whereas preictal discharges (PIDs) were preceded by pyramidal cell firing that depended solely on glutamatergic transmission^4^. Furthermore, IIDs spread from the SOZ to adjacent cortical regions approximately 11 s before seizure onset, when PIDs were restricted to the SOZ^4^. The low MSE score in the interictal period might reflect interneuron synchronization associated with the frequent IIDs in FCD type II. This may explain why interictal MSE scores were always the lowest in the SOZ. Another study in the human epileptic subiculum showed that IIDs and PIDs could only be distinguished by using combined intra- and extracellular recordings^24^. Therefore, we emphasize that low MSE scores can be a new marker that delineates the SOZ in FCD type II.

The rising MSE scores in the SOZ during preictal periods might reflect the breakdown of inhibitory interneuron hypersynchronization during interictal periods. Dynamic balance of excitation and inhibition breaks down during seizures, where the temporal correlation of excitatory and inhibitory neurons is dirupte^16^. We previously showed that the relative reduction in power of post-spike slow waves to spike-related HFOs (80–200 Hz) in the SOZ that occurs preictally is relevant for seizure initiation^5, 6^. The preictal decrease in the inhibition of slow waves enhanced the activity of principal neurons and the rebound excitatory synchronization of GABAergic networks, thus promoting the progression of seizure activity over the epileptogenic zone^44^. This preictal collapse of the balance between excitatory and inhibitory forces also led the epileptic network to assimilate the SOZ into the surrounding epileptogenic zone during preictal periods^15^. Despite this rising score in the SOZ during preictal periods, this trend was accompanied by lower scores in both the SOZ and RA-SOZ than outside the RA. We speculate that the spread of excitatory-dominant interneuron synchronization from the SOZ to the RA-SOZ might precipitate the seizure over the entire epileptogenic zone. If it were possible to identify a threshold in the preictal MSE score of SOZ gamma oscillations past which seizures always ensue, seizure initiation could be revealed by spatiotemporal analysis of MSE. However, further studies are required to validate this concept.

The ictal mechanisms of interneuron synchronization might be reflected by the significantly low ictal MSE scores, both in the SOZ and RA-SOZ, that correspond to the epileptogenic zone. A study in the rat model of epilepsy showed that interneuron became coherent with local field potential with gamma oscillations in the seconds before seizure onset, and that the increasing interneuron activity, rather than pyramidal cell activity, mediated ictal events^32^. During ictal periods, inhibitory synchronization is accompanied by an increase in extracellular potassium ([K + ]_o_), primarily via the K + -Cl- cotransporter (KCC2)^10^. This increase in [K + ]_o_ causes a decrease in GABA_A_ receptor-mediated chloride currents, which leads to a depolarizing block of inhibitory interneurons^31^. Consequently, excitatory interneuronal hypersynchronization spreads over the epileptogenic zone at the time of ictal onset^2^. The decreasing MSE score in the SOZ during ictal periods might entrain the epileptogenic zone into powerful hypersynchronization, thus precipitating the seizure. We expect that in a subset of patients with FCD type II, the epileptogenic zone can be determined as the boundary between regions in which the MSE score decreases and those in which it remains high, and that the timing of the decrease will coincide with the beginning of the ictal period.

Physiological HFOs in normal hippocampal ripples occur when inhibitory interneuron networks entrain pyramidal cells and other interneurons into rhythmic firing^45^. It has been reported that pathological HFOs may reflect pathologic neuronal synchronization in epileptic tissue that is induced by the normal physiological mechanisms of gamma-band HFO generation^23^. The areas with lower MSE scores during ictal periods might indicate more pathological HFOs along with different degrees of epileptiform hypersynchronization, which could be used to differentiate between pathological and non-pathological HFOs. The pathological HFOs cannot always be detected from EEG data because they constitute paroxysmal EEG activity^19^. Therefore, pathological HFOs can be more accurately found using the low MSE scores during the ictal period. Further studies will be necessary to clarify the correlation between low MSE scores in gamma oscillations and the appearance of HFOs during ictal periods. Alongside our MSE analysis, magnetic resonance imaging (MRI), functional MRI, positron emission tomography, or magnetoencephalography (MEG) will also be necessary to further investigate the spatial configuration of the epileptogenic zone for both FCD type II and focal epilepsy provoked by lesions that are invisible by MRI.

**Figure 5:**
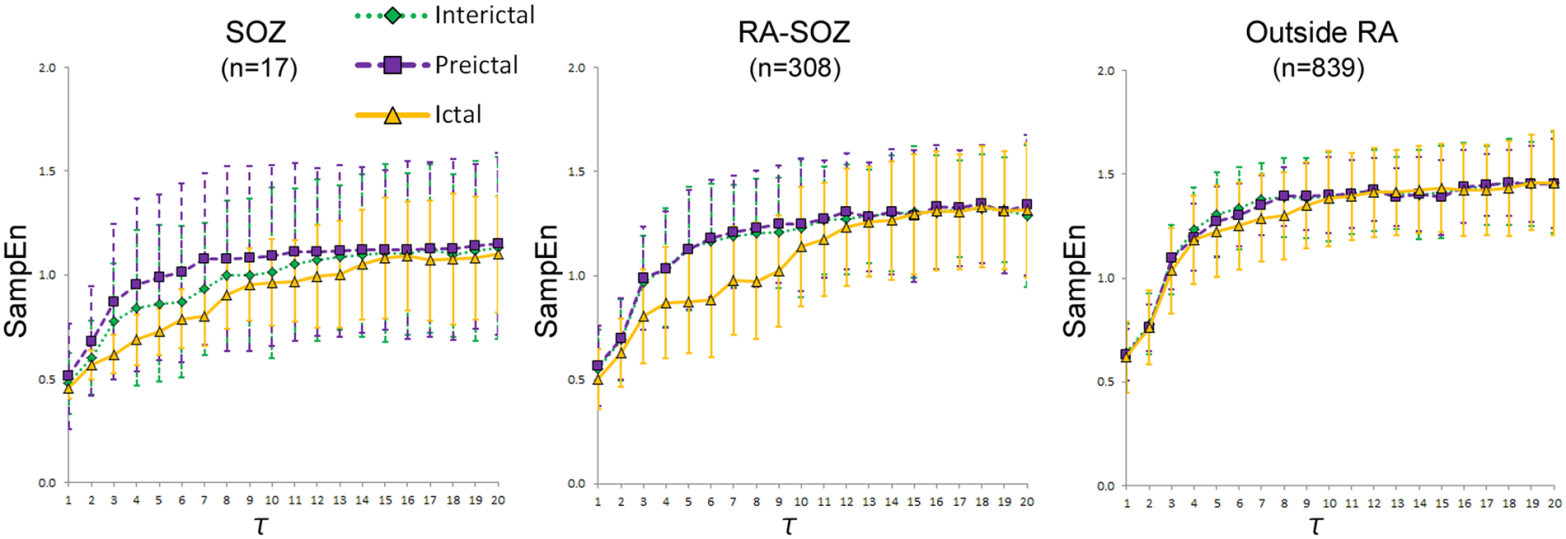
Multiscale entropy (MSE) curve with mean values and standard error of sample entropy (SampEn) over time scale factor τ = 1 to 20 among three periods for each area. n represents the number of electrodes analyzed for each area. SOZ, seizure onset zone; RA-SOZ, resection area outside the SOZ; Outside RA, outside resection area. In τ = 3 to 7 corresponding to the gamma frequency (28.6–66.7 Hz), the MSE curves in the SOZ apparently differentiated among three periods, and the MSE curves in the RA-SOZ apparently differentiated between ictal period and the other two periods.

This study has some limitations. First, as shown in Fig. 5, even in τ = 8 to 12 corresponding to the beta band (17–25 Hz), the MSE curves in the SOZ differentiated among three periods, and MSE curves in the RA-SOZ differentiated between ictal period and the other two periods. The usage of the full “discriminative band” ranging from beta to gamma band would enhance or reduce the ability of SOZ and RA identification. Further detailed analysis in each scale factor τ would be needed to address this issue. Second, the interictal variability could have addressed with various convinced ways. We randomly selected three interictal segments during non-REM sleep. We confirmed that there were no significant differences between the MSE score variances (p > 0.05) and the MSE score (p > 0.05) among them using Bartlett’s test and one-way analysis of variance. Further studies would be required to offer more accurate interictal MSE scores as the reference data.

Nonlinear EEG analyses in epilepsy have demonstrated that they can detect seizure onset using artificial neural network model^46^, and the strong indications of nonlinear deterministic structure are associated with epileptiform activity^47^. In line with these evidences, the activity of epileptic neuronal networks can be depicted as such nonlinear dynamical process regulated by synchronous or desynchronous mechanisms across multiple neuronal populations. In the present study, we used MSE analysis using the sample entropy (SampEn), which is a robust estimator for evaluating dynamical EEG activity^48^, to measure nonlinear-deterministic process with the regularity of gamma oscillations related to interneuron synchronization^36–38^. Our results support the evidence that the nonlinear dynamic processes certainly underlie epileptiform networks. This study for the first time revealed the spatiotemporal changes in MSE score derived from EEG gamma oscillations that occur in patients with FCD type II. The dynamics of the neuronal activity represented by these scores may explain the association between focal ictogenesis and interneuron networks. Thus, the spatiotemporal analysis of MSE in gamma oscillations can provide a reliable marker of the SOZ, the epileptogenic zone, and the timing of ictal onset in human focal epilepsy.

## Methods

### Patient Selection

We retrospectively analyzed 13 pediatric patients (8 males and 5 females) undergoing intracranial video-EEG monitoring for the surgical treatment of medically refractory focal epilepsy secondary to FCD type II between 2008 and 2013 at the Hospital for Sick Children in Toronto, Canada. The sites of electrode placement were individualized according to clinical history, seizure semiology, neuroimaging, and scalp EEG findings, as described previously^49^. All patients met the following criteria: (1) clearly visible cortical dysplastic lesions on MRI; (2) postsurgical histopathological diagnosis revealed FCD type II; and (3) postsurgical seizure outcome was rated as class I (completely seizure free; no auras) according to the International League Against Epilepsy (ILAE) classification system^50^. Patient characteristics are shown in Table 1. All Methods were performed in accordance with the approved guidelines by the Research Ethics Board at the Hospital for Sick Children. All patients or their families gave informed consent approved by the Research Ethics Board at the Hospital for Sick Children for study participation.

### Intracranial EEG Recordings

We implanted a subdural grid so as to cover the FCD lesions, as described previously^51^. The inter-electrode distances ranged from 8 to 10 mm (Ad-Tech, Racine, WI, U.S.A.). EEG data were acquired using a Harmonie system (Stellate, Montreal, PQ, Canada) with a sampling rate of 1 kHz. The recordings were performed referentially with the reference electrode placed in an inactive area that never registered epileptic discharge.

### Determination of the seizure onset zone (SOZ) and the resection area (RA)

The SOZ and RA were defined using standard methods^19, 21^. Briefly, the SOZ was determined by certified clinical neurophysiologists as the electrodes that recorded the earliest ictal-related low amplitude, fast activity. HFOs (>80 Hz) were also expressed in the SOZs of all patients. The SOZ was defined by one electrode in 9 patients and by two electrodes in 4 patients. The RA was delineated on the intraoperative photos by the integration of ictal and interictal EEG findings on the intracranial electrodes after intracranial video EEG, MRI and MEG findings, neuropsychological and neurological assessments. The RA including the SOZ and active interictal zone was proposed at the surgical resection. The RA in this paper was equal to the final resection margin.

### EEG Data Selection

For each patient, we selected one typical habitual seizure that lasted at least 30 s (55.3 ± 26.1 s) and extracted three 20-s epochs – one interictal, one preictal, and one ictal. Interictal epochs comprised the 20 s of activity during non-REM sleep, separated by at least 1 h from any seizure onset. Preictal epochs comprised the 20 s of activity immediately preceding electrographic seizure onset. Ictal epochs comprised the 20 s of activity immediately following the beginning of the rhythmic ictal pattern. Since focal seizures are typically very similar, the preictal and ictal EEG data can be regarded well-defined. This is in contrast to the 20 s of interictal EEG per patient. Therefore we randomly selected two other 20 s interictal epochs (separated from seizure activity by at least 1 h), compared with the first interictal epoch using Bartlett’s test and one-way analysis of variance. All selected epochs were carefully inspected to ensure that they did not contain any significant artifacts.

### MSE Analysis of Gamma Oscillations

MSE analysis quantifies the regularity of EEG series at multiple time scales^37^. Small MSE scores reflect regular EEG rhythmicity. Here we calculated the MSE scores using SampEn^48^. The original EEG series *X* = {*X*_1_,* × *_2_, … *X*_*N*_} is coarse-grained by the time scale factor (τ) that is set as the width of non-overlapping windows. The other coarse-grained time series *Y* ^(τ)^ = {*Y*_1_, *Y*_2_, … *Y* $\frac{N}{{\rm{\tau }}}$} is defined as

1 $${Y}_{j}^{({\rm{\tau }})}=\frac{1}{{\rm{\tau }}}\sum _{{\rm{i}}=({\rm{j}}-1){\rm{\tau }}+1}^{j{\rm{\tau }}}{X}_{i},\quad 1\le j\le \frac{N}{{\rm{\tau }}}$$

The time series *Y* ^(1)^ is identical to the original time series, which represents a short-range temporal scale, whereas higher τ values represent longer temporal scales. The MSE score with τ (MSE^(τ)^) was calculated for each series *Y* ^(τ)^. MSE^(τ)^ depends on three parameters: N (the total number of data points), m (the number of the consecutive data points to be compared), and r (a noise threshold for measuring the consistency of the time series). MSE^(τ)^ in the coarse-grained time series *Z* = {*Z*_1_, *Z*_2_, … *Z*_*N*_} is defined as

2 $$\begin{array}{c}MS{E}^{({\rm{\tau }})}(N,m,r)={\mathrm{log}}_{e}[{C}_{m+1}(r)/{C}_{m}(r)]\\ {\rm{where}}\,{C}_{m}(r)=\tfrac{\{{\rm{number}}\,{\rm{of}}\,\mathrm{pairs}\,({\rm{i}},{\rm{j}})\mathrm{with}\,|{Z}_{i}^{m}-{Z}_{j}^{m}| < r,i\ne j\}}{\{{\rm{number}}\,{\rm{of}}\,{\rm{all}}\,{\rm{probable}}\,{\rm{pairs}},{\rm{i}}.{\rm{e}}.,(N-m+1)(N-m)\}}.\end{array}$$

*Z* ^*m*^ is a vector of m members time series of (N – m) length, and $|{Z}_{i}^{m}-{Z}_{j}^{m}|$ denotes the distance between points ${Z}_{i}^{m}$ and ${Z}_{j}^{m}$ in the space of dimension m (for details of the SampEn algorithm see Richman and Moorman, 2000)^48^. Thus, the MSE score is the negative natural logarithm of the conditional probability that at all data points (N), two sequences similar to each other for the first m points remain similar also at the next point (m + 1).

All EEG data were filtered using a 60-Hz notch filter and down-sampled to 200 Hz using the EEGLAB toolbox (http://sccn.ucsd.edu/eeglab) to efficiently analyze the gamma frequency ranges. Figure 5 shows the MSE curves with mean values and standard error of SampEn over time scale factor τ = 1 to 20. Given the sampling rate of 200 Hz, the time scale factor τ = 3 to 7 approximately corresponded to the gamma frequency (30–70 Hz), because

3 $$\frac{200\,Hz}{3}=66.7\,Hz,\quad \frac{200\,Hz}{7}=28.6\,Hz.$$

Accordingly, we defined the MSE score for gamma oscillations as the averaged value of MSE scores with τ = 3 to τ = 7, which were obtained by the following formula:

4 $${\rm{MSE}}\,{\rm{score}}\,{\rm{for}}\,{\rm{gamma}}\,{\rm{oscillations}}=\frac{1}{5}\sum _{{\rm{\tau }}=3\,}^{7}\,MS{E}^{({\rm{\tau }})}$$

Accordingly, when MSE scores in the gamma band are low, the regularity of neurophysiological activity at timescales corresponding to the gamma band is high. Previous studies have shown that values of m = 2 and r = 0.2 provide good statistical validation for MSE analysis of EEG data^41, 42^. For each selected 20-s epoch, we calculated MSE scores with N = 4000 (i.e., 20 s × 200 Hz), m = 2, and r = 0.2, and created topographic maps by superimposing the calculated MSE scores onto an intraoperative photograph of each patient’s brain surface. These procedures were performed using a custom program written in MATLAB (The MathWorks, Version 8.5, Natick, MA, U.S.A).

### Statistical Analysis

We evaluated differences in MSE scores during the interictal, preictal, and ictal periods of each patient. Across patients, we used the Steel-Dwass test^51, 52^, as a nonparametric multiple comparison procedure because the obtained MSE scores had non-Gaussian distributions, to evaluate the differences in MSE scores among regions (SOZ, RA-SOZ, and outside the RA) during the three periods. Statistical analyses were performed with MATLAB and Microsoft Excel 2010 (Microsoft Corp., Seattle, WA, U.S.A.).
